# Mental activities after dinner increase cigarettes consumption

**DOI:** 10.1038/s41598-021-81978-y

**Published:** 2021-01-28

**Authors:** Xuechan Yu, Yiming Yu, Hongying Ma, Zhongbo Chen, Zaichun Deng

**Affiliations:** Department of Pulmonary and Critical Care Medicine, The Affiliated Hospital of Medical School of Ningbo University, No. 247, Renmin Road, Jiangbei District, Ningbo, Zhejiang Province China

**Keywords:** Cellular immunity, Chronic inflammation

## Abstract

Tobacco smoking is the main risk factor for many diseases such as chronic obstructive pulmonary disease as well as lung cancer and cigarettes. Smokers usually keep continuing to smoke during their mental activities in the evening between dinner and sleep time on work days. So it is critical to elucidate the relationship between cigarettes daily consumption and mental activities after dinner. A survey designed by ourselves was finished among 369 patients who came to our clinic for smoking cessation. Age, gender, BMI, cigarettes consumption were recorded and analyzed. Statistically, Pearson correlation test and general linear model test were used. Compared to ≤ 40 years’ group, patients with mental activities after dinner consumed more cigarettes than those without mental activities (22.80 ± 10.86 vs. 30.88 ± 18.69, *P* value < 0.001). The Pearson correlation test showed no interact effects on age and BMI, and general linear model test showed that the cigarettes numbers between smokers with mental activities after dinner and smokers without mental activities after dinner are different (*P* value < 0.001). Mental activities from dinner finish to sleep time increase cigarettes consumption. It provides evidence that mental thinking activities after dinner is a risk factor of tobacco using.

## Introduction

Cigarette smoking is prevalent as a health risk behavior in the general population and it is the main risk factor which has been triggered various malignant cancers^1^ as well as chronic obstructive lung disease^2^. We can learn from paper that unhealthy behaviors or risk factors lead to smokeing more frequently^3^. Cigarettes smokers usually keep continuing to smoke during their mental activities in the evening between dinner and sleep time on work days such as play video games, gathering gambling, study, long-time work, search information from the Internet and so on. These studies showed that gambling and playing video games can cause more consumption of tobacco^4,5^. However, these data don’t represent all activities with mental thinking. So it is critical to elucidate the relationship between cigarettes daily consumption and mental activities after dinner.

## Results

### Survey results in two different age groups

From Table 1, no significantly differences in age, gender, and BMI in two age groups with or without habit of mental activities. We can draw conclusion that no cigarettes number differencess in ≤ 40 years’ group whether have mental thinking after dinner (20.39 ± 8.12 vs. 21.96 ± 11.91, *P* value = 0.342). Patients with mental activities after dinner consumed more cigarettes than those without mental activities (22.80 ± 10.86 vs. 30.88 ± 18.69, *P* value < 0.001).

**Table 1:** Survey results in two groups.

	≤ 40 years (n = 166)			> 40 years (n = 203)		
	No mental activities after dinner (N = 71)	Mental activities after dinner (N = 95)	*P* value	No mental activities after dinner (N = 128)	Mental activities after dinner (N = 75)	*P* value
**Age, years**						
Mean ± SD	34.15 ± 4.96	32.72 ± 4.96	0.066	51.72 ± 7.72	49.65 ± 7.72	0·067
**Gender, n**						
Male	63	84	0.576	120	73	0.215
Female	8	11		8	2	
**BMI**, **Kg/m**^2^						
Mean ± SD	23.39 ± 3.18	22.40 ± 3.29	0.053	24.10 ± 9.26	23.82 ± 2.98	0.801
**Cigarettes consumption (per day)**						
Mean ± SD	20.39 ± 8.12	21.96 ± 11.91	0.342	22.80 ± 10.86	30.88 ± 18.69	< 0.001

### Further analysis in > 40 years’ group

From Table 2, further evidences confirmed by Pearson correlation test show that there are no interactive effects on age and BMI to daily smoking cigarettes number (Age 0.085, *P* value  = 0.226; BMI 0.022,* P* value  = 0.752). General linear model test improved that the cigarettes numbers between smokers with mental activities after dinner and smokers without mental activities after dinner are statistically different (*P* value  < 0.001).

**Table 2:** Pearson correlation and general linear model test in > 40 years’ group.

	Pearson correlation	*P* value	General linear model test * P* value
Age	0.085	0.226	0.067
BMI	0.022	0.752	0.801
Cigarettes consumption	1		< 0.001

## Discussion

Tobacco consumption with mental activities during dinner finish to sleep is more common on work days. Therefore, it is critical to further confirm that whether mental thinking activities can cause more cigarettes smoked. Self-designed survey finished by patient went to smoking cessation clinic. From analyzed results, we found that more daily cigarettes number consumed with mental thinking after the age of 40.

Considering difference in occupation and professional career between two age groups, social stress in various job types may cause different cigarettes consumption. It is similar to the researches published before^6,7^ and this is the reason why patients classified.

Many studies in the past have mentioned an increase in smoking when smokers engage in mental thinking. After exhausting daytime work, mental behaviors after dinner without rest may increase tiredness. Meanwhile, nicotine provided from tobacco produce dopamine for getting the brain’s attention to activities so that cigarettes consumption is increased potentially more than patients’ with no mental thinking. When people doing mental activities, dopamine level will increase to help brain deal with problems^8^. Therefore, we can understand smokers preferring choose tobacco at night. We also found the same explanation from the paper published by Gray et al^9^. Gambling, play video games, work at night are all regarded as mental thinking activities, and our data showed that tobacco amount increased. Likely, these studies and researches showed the same results^4,5,10–12^.

There is the difference compared to previous study. We inferred from study^13^ that age is a factor of cigarettes smoking and nicotine dependence. Unlikely, our tables showed there is no significance in two age groups. Because the age criteria and aim population for classify are completely different, so that Pearson correlation test was used for elimination of interaction effect, which ensured the data’s quality.

Our research also has limitations. Firstly, we didn’t collect about nicotine dependence information so it is not known whether mental activities can influence patients’ urge of nicotine. Secondly, only 75 patients with mental behavior in 40 years’ group increased daily cigarettes number, the results must be confirmed in a large study, which also contain other research content, including using urge to smoke, dependence and other craving questionnaires. Thirdly, we regretfully neglected to record what kind of activity influenced most in these patients. Fourthly, following intervention study of mental activities during cessation is necessary which may help patients increase conscious of avoiding potential smoking behavior.

In conclusion, mental activities during dinner finish to sleep time increase cigarettes consumption. Our study adds to the accumulating evidence that mental thinking activities after dinner is a risk factor of tobacco smoking. And our data provides suggestion that avoiding this behavior may raise the possibility during nicotine cessation.

## Materials and methods

### Materials and survey

A self-designed questionnaire survey conducted by every patient who went to the clinic for smoking cessation in The Affiliated Hospital of Medical School of Ningbo University from April 2010 to January 2020. Our study and survey designation approved by the Ethics Committee of the Affiliated Hospital of Medical school of Ningbo University (Ningbo, China). We chose smokers over the age of 20 who came to the clinic for smoking cessation. Mental activities were defined that gambling, overtime work, play video games, studying or other mental thinking activities. Smokers with mental activities more than 1 h after dinner before sleep were included in the study as the main study comparison factor. Smokers with mental activities on weekend can not be excluded. In China, different occupations have different need, people often work on weekends, or even no rest. Considered about age as the factor which influenced the cigarettes daily consumption, state of life and career with different mental distress and concerns, patients were divided into two different age groups (≤ 40 years’ group and > 40 years’ group). Informed consent contained from all patients before finish the questionnaire. All methods were carried out in accordance with NCCN Clinical Practice Guidelines in Oncology of Smoking Cessation^14^. Age (years), Gender, BMI (kg/m^2^), cigarettes consumption (per day) and mental activities after dinner (yes or no) were asked in the questionnaire. Questionnaire was shown in supplementary material.

### Statistical analysis

After exclusion of incomplete or incorrect information, the results of the questionnaire for 369 patients were included in the statistics. In each age group, patients were categorized according to whether have mental activities after dinner. Gender distribution was calculated by Fisher’s exact test. Mean ± standard deviation (SD) and *t* test were used in age, BMI, cigarettes consumption.

Pearson correlation examination was used in > 40 years’ group for further confirm that factors (age, BMI) wouldn’t make any effects in daily consumption of cigarettes. Variables (age, BMI, cigarettes consumption) were tested by the general linear model (no activity or any kind of mental activity).

The analyses were calculated by IBM SPSS Statistics 21.0 and* P* values < 0.01 were judged significantly.

### Ethical approval

Our study and survey designation approved by the Ethics Committee of the Affiliated Hospital of Medical school of Ningbo University (Ningbo, China). Informed consent was obtained from all patients before conducted the survey.

## Supplementary Information

Supplementary Information.

## Data Availability

Our data generated and analyzed during the study are available from the corresponding authors in the future research on reasonable request.
