# Supplementary material for: Mental activities after dinner increase cigarettes consumption
Source: Sci Rep. 2021 Jan 28;11:2405. doi: 10.1038/s41598-021-81978-y (PMC7843627; doi:10.1038/s41598-021-81978-y)
Supplement: Supplementary file 1 — Supplementary Information. [file 41598_2021_81978_MOESM1_ESM.docx]

**Mental activities after dinner increase cigarettes consumption**

Xuechan Yu^+^, Yiming Yu^+^, Hongying Ma, Zhongbo Chen^*^,Zaichun Deng^*^

Department of Pulmonary and Critical Care Medicine, The Affiliated Hospital of Medical School of Ningbo University, Ningbo 315010, China.

^*^**Correspondence to:**

^1^Zhongbo Chen

Department of Pulmonary and Critical Care Medicine,

The Affiliated Hospital of Medical School of Ningbo University,

No.247, Renmin Road, Jiangbei District

Ningbo, Zhejiang Province, China

Phone: 86-1377125910

Fax: 86-0574-87355381

Email: leonczb@163.com

^2.^Zaichun Deng

Department of Pulmonary and Critical Care Medicine,

The Affiliated Hospital of Medical School of Ningbo University

No.247, Renmin Road, Jiangbei District

Ningbo, Zhejiang Province, China

Phone: 86-15267835908

Fax: 86-0574-87355381

Email: dengzaichun@nbu.edu.cn

+ These authors contributed equally and both regarded as first authors in this study.

Phone：86-13429321045；Fax: 86-0574-87355381

**Questionnaire for smokers in clinic**

Date Name

Age (years)

BMI (Kg/m^2^)

Gender

Cigarettes daily consumption (per day)

More than 1 hour mental activities after dinner: yes no
